# Latent profile analysis of depressive symptoms among Chinese older adults with disabilities and associated factors

**DOI:** 10.3389/fpsyt.2026.1830637

**Published:** 2026-05-08

**Authors:** Xiumei Gao, Yu Han

**Affiliations:** 1 Department of Cardiology, the First Affiliated Hospital of Jinzhou Medical University, Jinzhou, China; 2 Department of Emergency Surgery, the First Affiliated Hospital of Jinzhou Medical University, Jinzhou, China

**Keywords:** anxiety, associated factors, depressive symptoms, latent profile analysis, older adults with disabilities

## Abstract

**Background:**

With population aging in China, the number of older adults with disabilities is increasing, and mental health problems, especially depressive symptoms, have become increasingly prominent. Examining latent profiles of depressive symptoms and their associated factors in this population may improve understanding of symptom heterogeneity and inform future screening and supportive care research.

**Methods:**

This study employed data from the 2018 Chinese Longitudinal Healthy Longevity Survey (CLHLS). Latent profile analysis (LPA) was used to identify latent profiles of depressive symptoms among older adults with disabilities. Subsequent analyses, including univariate and multinomial logistic regression, were conducted to examine factors associated with depression profile membership.

**Results:**

The 1, 346 older adults with disabilities were classified into three latent depressive symptom profiles: low symptom burden (24.4%), moderate symptom burden (55.5%), and high symptom burden (20.1%). In univariate analyses, all variables except serious illness differed significantly across profiles. In multinomial logistic regression analyses, compared with the low-level profile, membership in the moderate-level profile was associated with MMSE, anxiety, marital status, education level, co-residence status, residential area, drinking, smoking, exercise, self-rated health, and activity limitations. Membership in the high-level profile was associated with MMSE, anxiety, gender, age, marital status, education level, co-residence of the interviewee, drinking, and self-rated health. Compared with the moderate-level profile, membership in the high-level profile was associated with MMSE, gender, marital status, residential area, drinking, and activity limitations.

**Conclusions:**

Distinct depressive symptom profiles were identified among older adults with disabilities, and several demographics, health-related, and psychosocial factors were associated with profile membership. These findings may inform future mental health screening and provide a basis for further longitudinal and intervention research.

## 1 Introduction

Population aging has become a global phenomenon, significantly impacting societal progress and economic vitality across nations. According to the World Health Organization, the global population aged 60 years and older reached nearly one billion in 2019. This number is expected to more than double to 2.1 billion by 2050 (1). In China, a nation of over 1.4 billion people and a leading global economy, a significant demographic shift is anticipated by 2050. The country is expected to have 400 million adults aged 65 years and older, including 150 million aged 80 years and above (2). Population aging and increased life expectancy are contributing to a growing number of older adults, including those with disabilities and those living alone (3). By 2020, the Chinese Center for Disease Control and Prevention reported that the number of older adults with disabilities had risen to 52.71 million, with projections suggesting this figure will swell to 77 million within the next decade, particularly in rural regions (4).

Mental health is increasingly being recognized as a core component of overall health. In later life, it is important to distinguish between depressive symptoms, screen-positive probable depression identified by screening tools, and clinically diagnosed depressive disorder, as these constructs are related but not interchangeable. Depression, particularly clinically diagnosed depressive disorder, is a major contributor to the global burden of mental illness. Previous studies have reported that the prevalence of clinically diagnosed depression among older adults is 1.6% in the United States and 2.7% in China (5, 6). However, these estimates should not be interpreted as the prevalence of depressive symptoms more broadly. Compared with older adults without disabilities, those with disabilities may experience greater functional limitation, chronic pain, frustration, social isolation, and reduced independence, all of which may be associated with higher levels of anxiety and depressive symptoms. A survey of adults aged 50 years and older across six countries with different income levels found a positive association between impairments in Basic Activities of Daily Living (BADL) and Instrumental Activities of Daily Living (IADL) and depressive symptoms (7). In Ireland, a long-term study of the elderly revealed that depression was associated with BADL/IADL disability (8). Multiple longitudinal studies have further shown that impairments in activities of daily living (ADL) are associated with depressive symptoms over time, with evidence suggesting a bidirectional relationship (9, 10). A cross-sectional study of 5, 863 elderly individuals in China further confirmed the association between depressive symptoms and ADL impairments (11). In addition, longitudinal trajectory studies have suggested that more severe ADL impairment may be associated with a greater likelihood of subsequent depressive symptoms (12). Among older adults with disabilities, depressive symptoms may be associated with poorer overall well-being, including reduced appetite, poorer weight maintenance, lower energy levels, and impaired immune function, and may coexist with worse quality of life and higher mortality risk.

Hou and Zhang utilized the Chinese Longitudinal Healthy Longevity Survey (CLHLS) to track 1, 831 elderly individuals living alone, identifying latent depressive symptom profiles categorized as low-level, moderate-level, and high-level (13). Curran et al. used data from the Irish Longitudinal Study on Ageing (TILDA, 2009-2011) to investigate gender-specific symptom subtypes of anxiety and depression in later life, classifying them into four groups: ‘low’, ‘comorbidity’, ‘anxiety and subthreshold depression’, and ‘anxiety’ only (14). In Spain, Pérez-Belmonte et al. employed latent class analysis to differentiate depression in the elderly into three distinct subgroups: psychosomatic, melancholic, and anhedonia (15). Furthermore, a prospective multinational cohort study involving 69, 066 participants used latent growth mixture modeling to understand the trajectories of depressive symptoms in older adults, classifying them into four categories: constantly low, constantly high, increasing, and decreasing (16). More recent studies have continued to show that late-life depression is heterogeneous rather than uniform. For example, Taiwo et al. identified distinct latent symptom profiles in community-dwelling older adults with late-onset depressive symptoms, with amotivation and anhedonia emerging as salient features in some subgroups (17). In addition, Solomonov et al. reported three clinical subtypes of late-life depression that differed not only in symptom burden but also in social support, disability, and subsequent response to psychosocial interventions (18). More recently, Liu et al. identified four latent depressive symptom profiles among older adults with chronic diseases, further suggesting that depressive heterogeneity in later life may vary across health contexts (19). Together, these studies support the value of person-centered approaches for clarifying clinically meaningful differences in depressive symptom patterns among older populations.

Although previous studies have identified heterogeneity in depressive symptoms among older adults, the mechanisms underlying such patterns in disabled older adults remain insufficiently explained (20). In this population, depression should not be viewed solely as an emotional response to disability, but also as a multidimensional mental health outcome shaped by age-related neurobiological vulnerability and psychosocial stress (21, 22). From a neurobiological perspective, late-life depression has been associated with structural and functional alterations in brain regions involved in mood regulation, cognitive control, and stress processing, particularly the prefrontal cortex, hippocampus, and anterior cingulate cortex (23). For disabled older adults, the combined burden of functional decline, chronic disease, pain, and reduced autonomy may further interact with these age-related brain changes, thereby contributing to different depressive symptom patterns.

In addition, the mental health of disabled older adults can be better understood within established models of aging and mental health (20). The stress-vulnerability perspective suggests that age-related physiological decline may increase susceptibility to depression when individuals are exposed to persistent stressors such as disability, social isolation, and reduced independence (24). Meanwhile, the biopsychosocial model emphasizes that depressive symptoms in later life arise from the dynamic interplay of biological changes, psychological adaptation, and social environment (21). Therefore, depression profiles in disabled older adults likely reflect not only differences in symptom severity, but also differences in underlying cognitive, emotional, and social vulnerability.

Methodologically, several approaches may be used to examine heterogeneity in depressive symptoms, including factor analysis, traditional clustering methods, and person-centered mixture models. In the present study, latent profile analysis (LPA) was preferred because the aim was not only to examine correlations among CESD-10 items, but also to identify subgroups of older adults with similar overall symptom presentations. Factor analysis is useful for describing the dimensional structure of symptoms at the variable level, whereas LPA is more suitable for detecting between-person heterogeneity at the individual level. Compared with conventional clustering approaches, LPA also provides model-based fit indices and posterior classification probabilities, which may support a more transparent evaluation of profile solutions. Therefore, the use of LPA in the present study should be understood as a person-centered descriptive approach that complements, rather than replaces, variable-centered methods such as factor analysis.

Based on this perspective, the present study used latent profile analysis to identify latent subgroups of depressive symptom burden among Chinese older adults with disabilities and to examine factors associated with profile membership. Given that the retained profiles may primarily reflect graded differences in symptom burden, the present analysis should be interpreted as describing between-person heterogeneity in depressive symptoms rather than establishing definitive clinical subtypes. By linking this heterogeneity with aging-related mental health theories and potential neurocognitive vulnerability, this study seeks to provide a more conceptually grounded understanding of depression in this population and to provide a basis for future research on mental health assessment and support in older adults with disabilities.

## 2 Materials and methods

### 2.1 Sample

**Figure 1:**
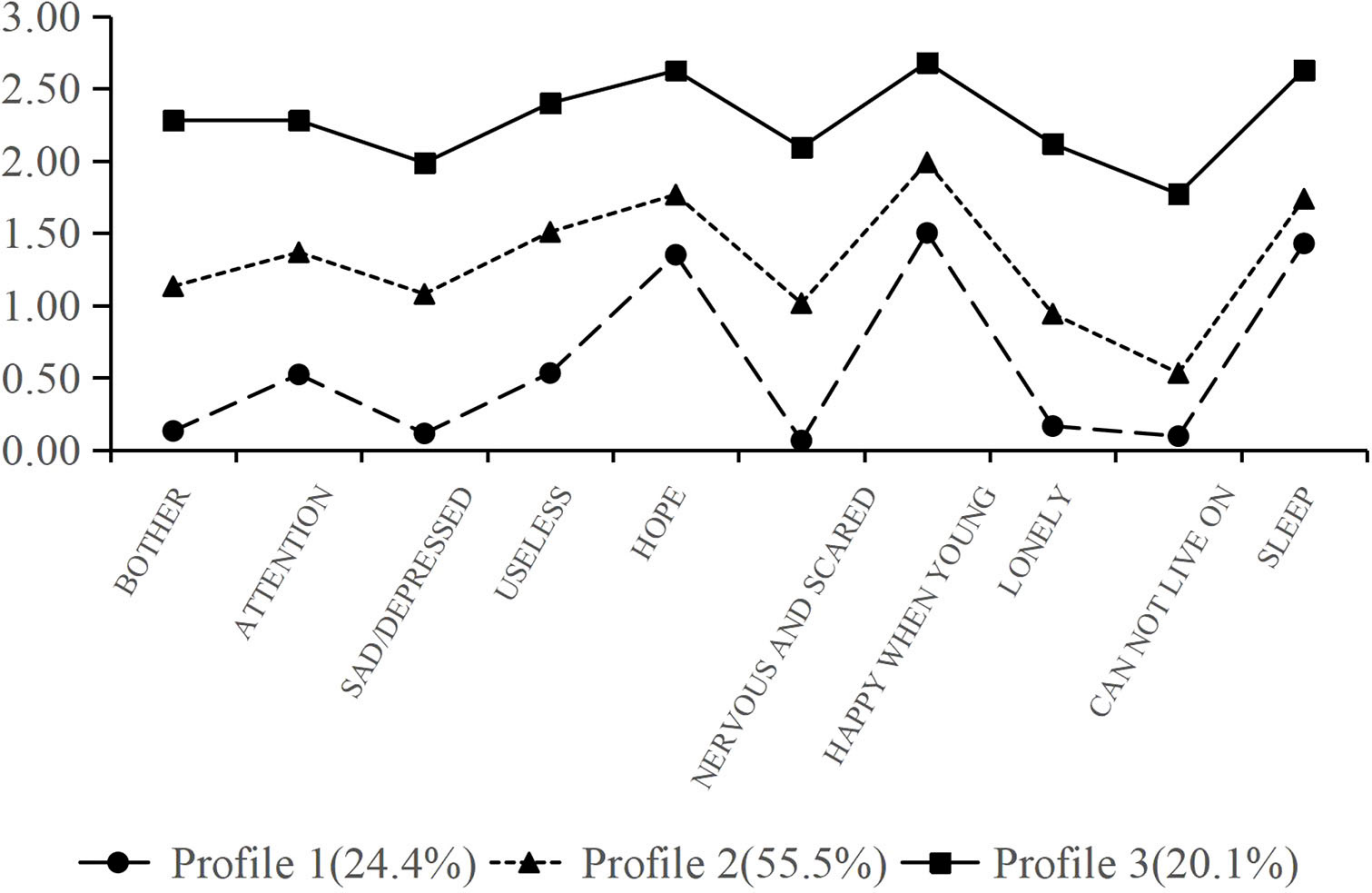
Flow diagram of sample selection.

This study used data from the CLHLS, available at https://opendata.pku.edu.cn/, which is administered by the Chinese Center for Disease Control and Prevention in collaboration with Peking University’s Center for Healthy Aging and Development Studies. CLHLS is a nationwide, multi-stage longitudinal survey conducted every two to three years since 1998. The survey covers 23 provinces across China, providing broad geographic coverage and national representativeness. From 1998 to 2018, CLHLS conducted eight follow-up surveys, accumulating interviews with over 113, 000 households. The survey assesses the health of the elderly from social, behavioral, environmental, and biological perspectives, providing a solid foundation for academic studies and public policy analyses. This database is widely used in aging research because of its rich content and broad applicability. This study utilized the latest cross-sectional data released in 2018, comprising 15, 874 survey responses. Participants were eligible for inclusion if they were aged 65 years or older, met the operational definition of disability based on BADL/IADL, and had data available for the main study variables. Individuals were excluded if they had missing responses on the CESD-10, lacked information required to determine disability status, had missing data on key covariates used in the analysis, or did not provide informed consent. Therefore, the final analytical sample consisted of 1, 346 older adults with disabilities (**Figure 1**). In the present study, a complete-case approach was used; that is, only participants with sufficient information on the study variables were retained for analysis. Because this was a secondary analysis of a public dataset, the reasons for nonresponse or missing data at the individual-item level could not be fully determined. To improve transparency, the category previously labeled as “other missing information” in **Figure 1** should be understood as missingness in one or more variables required for sample definition or subsequent analyses.

### 2.2 Disability assessment

In this study, disability was identified using the BADL and IADL items available in the 2018 CLHLS. The BADL scale includes six domains: bathing, dressing, toileting, indoor mobility, continence, and eating. The IADL scale evaluates eight daily activities, including visiting neighbors, shopping, cooking, washing clothes, walking, lifting weight, squatting, and using public transportation. Each item is rated on a 3-point scale, with higher scores indicating greater dependence. Participants were classified as having disability if they were reported as being completely dependent in at least one BADL or IADL item. This operational definition was adopted to identify older adults with clear functional dependence in basic or instrumental daily activities and is consistent with previous studies that have defined disability on the basis of dependence in any BADL or IADL item in Chinese older populations (25, 26). However, BADL and IADL reflect different levels of functional loss, with IADL limitations often indicating earlier or milder decline and BADL dependence reflecting more severe impairment (27). Therefore, the present definition should be understood as a broad screening criterion for functional disability rather than as implying a fully homogeneous disability group. In the 2018 CLHLS, the BADL/IADL measures demonstrated good internal consistency, with a Cronbach’s α of 0.818 (28).

### 2.3 Measurement

#### 2.3.1 Demographic information

Variables included gender, age, marital status, education level, co-residence status, residential area, drinking, smoking, exercise, self-rated health, and activity limitations.

#### 2.3.2 Depression

Since 2018, the CLHLS has used the 10-item Center for Epidemiologic Studies Depression Scale (CESD-10) (29) to assess depressive symptoms among older adults. This scale comprises 10 items and uses a four-point response format to assess respondents’ psychological states during the preceding week. Of these items, seven reflect potential negative psychological states, while three indicate positive psychological states. Responses indicating persistent negative psychological states are scored as 3, whereas responses indicating infrequent or absent negative psychological states are scored as 0. Conversely, responses indicating persistent positive psychological states are scored as 0, while those indicating infrequent or absent positive psychological states are scored as 3. The total CESD-10 score ranges from 0 to 30, with higher scores indicating greater depressive symptom severity. Among Chinese older adults, the CESD-10 is extensively used for assessing and screening depressive symptoms due to its high reliability and validity, demonstrated by a Cronbach’s α coefficient of 0.813 in this study. In the present study, the CESD-10 was used to assess the severity and patterning of self-reported depressive symptoms. It is a screening instrument and does not provide a clinical diagnosis of depressive disorder. For item-level interpretation, higher scores on all CESD-10 items were treated as indicating greater depressive symptom burden after coding; thus, for positively worded items, a higher score reflects reduced positive affect or reduced hopefulness rather than a more positive emotional state.

#### 2.3.3 Anxiety

The CLHLS project utilized the GAD-7 (30) to measure anxiety levels in older adults. This self-report scale assesses symptom frequency over the past two weeks and effectively screens for generalized anxiety disorder and its severity. The seven survey items are scored using a 4-point Likert scale, with responses ranging from never (0) to almost every day (3). Overall anxiety level was assessed using a total score ranging from 0 to 21, where higher values are associated with increased symptom severity. For descriptive purposes, participants with scores below 5 were classified as having no anxiety symptoms, whereas those with scores of 5 or higher were classified as having anxiety symptoms. The scale demonstrates high internal consistency, with a Cronbach’s α coefficient of 0.918, indicating strong reliability. In addition, the scale is valid and practical in the assessment of anxiety in Chinese older adults and has been widely used in several studies (31). In this analysis, the scale yielded a high internal consistency, denoted by a Cronbach’s α of 0.919.

#### 2.3.4 MMSE

The Mini-Mental State Examination (MMSE) stands as an internationally recognized instrument for assessing cognitive status (32), and its Chinese version plays an important role in cognitive assessment in China (33). Within the CLHLS framework, the MMSE evaluates cognitive function across five domains: orientation, registration, attention or calculation, recall, and language. Comprising 24 items, the MMSE is routinely employed to assess cognitive functioning. For item 6 of the MMSE, participants receive 1 point per food item named, up to a maximum of 7 points for correctly identifying over seven items. The remaining 23 questions are scored dichotomously, with participants receiving a score of 1 for correct answers and 0 otherwise. Scores on the Chinese MMSE range from 0 to 30 points, with higher scores indicating better cognitive function. Cronbach’s α values for the subscales assessing orientation, registration, attention/calculation, recall, and language abilities were 0.536, 0.712, 0.784, 0.677, and 0.729, respectively. The total Cronbach’s α coefficient, at 0.831, suggests a satisfactory level of internal consistency across the scale’s items.

### 2.4 Statistical analysis

Data management was performed using SPSS 25.0, and statistical analyses were conducted using SPSS 25.0 and Mplus 8.1. A two-tailed test with P < 0.05 was considered statistically significant.

#### 2.4.1 Common method bias assessment

In this study, many variables were collected using self-report questionnaires, which may introduce common method bias. Harman’s single-factor test (34) was used as a preliminary diagnostic approach. Exploratory factor analysis (EFA) was performed on all questionnaire items without rotation. If two or more factors were extracted and the first factor explained less than 40% of the total variance, serious common method bias was considered less likely. In particular, this procedure cannot fully address potential bias related to self-report measures, shared measurement context, or the cross-sectional design of the study.

#### 2.4.2 Descriptive analysis

Data were analyzed using SPSS 25.0. Initially, normality tests were conducted. Normally distributed measurement data were presented as mean ± standard deviation (M ± SD), while non-normally distributed data were expressed as median (M) and interquartile range (IQR). Frequency and percentage were used to summarize count data.

#### 2.4.3 Latent profile analysis

In this study, the 10 CESD-10 items were entered as profile indicators to identify latent subgroups of depressive symptoms among older adults with disabilities. LPA was conducted in Mplus 8.1 by fitting models with one to five latent profiles. Model fit was evaluated using Akaike’s information criterion (AIC), Bayesian information criterion (BIC), adjusted Bayesian information criterion (aBIC), entropy, the Lo-Mendell-Rubin likelihood ratio test (LMRT), and the bootstrap likelihood ratio test (BLRT). Lower AIC, BIC, and aBIC values indicate better relative fit, whereas entropy values closer to 1.0 indicate clearer classification. Significant LMRT and BLRT values suggest that a model with *k* profiles fits better than a model with *k − 1* profiles.

In selecting the optimal model, we did not rely on a single fit index. Instead, model selection was based on an overall consideration of statistical fit, classification quality, parsimony, profile size, and substantive interpretability. Because information criteria in mixture models often continue to improve as additional profiles are added, smaller AIC/BIC/aBIC values alone were not considered sufficient evidence for retaining more complex solutions. In the present study, profile solutions including classes with very small proportions were interpreted cautiously, because such classes may reflect sample-specific splitting of broader groups rather than stable and clinically meaningful subpopulations. Following common practice in mixture modeling, classes representing less than approximately 5%–10% of the sample were considered potentially unstable and were therefore evaluated with particular caution.

The final retained model was required to show acceptable classification quality, no excessively small classes, and a profile structure that was both clinically interpretable and distinguishable. In addition, the identified profiles were interpreted with attention to whether they reflected qualitatively different item-response configurations or primarily graded differences in overall depressive symptom severity.

#### 2.4.4 Univariate and multivariable analyses

Latent profile membership identified by LPA was treated as the dependent variable. Group differences were first examined using the chi-square test for categorical variables and the Kruskal-Wallis test for continuous variables. Variables with P < 0.05 in the univariate analyses were entered into a multinomial logistic regression model to examine factors associated with profile membership among older adults with disabilities. The adequacy of profile size for subsequent regression analysis was considered before model fitting. In the retained three-profile solution, the smallest class included 271 participants (20.1% of the sample), which was considered sufficient for multinomial comparisons.

## 3 Results

### 3.1 Common method deviation test

Common method deviation was assessed using Harman’s single-factor approach. EFA identified nine factors with eigenvalues greater than 1, and the first factor explained 13.470% of the total variance, which was below the conventional 40% threshold. This finding suggests that severe common method bias was not indicated by this preliminary test. However, because Harman’s single-factor test is limited in sensitivity, the possibility of residual method bias related to self-report measures and the cross-sectional design cannot be fully excluded.

### 3.2 General demographic characteristics

Female participants accounted for 57.4% of the sample. Most participants were aged between 65 and 95 years. In addition, 73.3% of participants lived with household members. Detailed demographic information is provided in **Table 1**.

**Table 1:** Demographic characteristics of participants.

Characteristics	N	%/M ± SD
Gender		
Male	574	42.6
Female	772	57.4
Age		
65-75	419	31.1
76-85	427	31.7
86-95	308	22.9
≥96	192	14.3
Marital status		
No spouse	727	54.0
Have a spouse	619	46.0
Education level		
None	608	45.2
1-6	411	30.5
≥7	327	24.3
Co-residence status		
With household member(s)	987	73.3
Alone	288	21.4
In an institution	71	5.3
Residential area		
City	463	34.4
Town	405	30.1
Rural	478	35.5
Drinking		
No	1123	83.4
Yes	223	16.6
Smoking		
No	1125	83.6
Yes	221	16.4
Exercise		
No	709	52.7
Yes	637	47.3
Self-rated health		
Good	607	45.1
Fair	509	37.8
Bad	230	17.1
Serious illness		
None	869	64.6
≥1	477	35.4
Activity limitations		
Strongly limited	120	8.9
Limited	473	35.1
Not limited	753	55.9
Anxiety (score, M ± SD)	—	1.45 ± 2.83
MMSE (score, M ± SD)	—	24.58 ± 5.74

### 3.3 Latent profile analysis of depressive symptom profiles in older adults with disabilities

#### 3.3.1 Results of latent profile analysis

The fit indices for the competing latent profile models are shown in **Table 2**. As the number of profiles increased from one to five, AIC, BIC, and aBIC continued to decrease, and LMRT and BLRT remained statistically significant. However, these improvements were interpreted cautiously, because in mixture modeling information criteria often continue to decline as model complexity increases. Therefore, the final model was selected by considering not only statistical fit, but also entropy, profile size, parsimony, and substantive interpretability.

**Table 2:** Indicators for latent profile of depression in disabled elderly individuals.

Profile	k	Likelihood	AIC	BIC	aBIC	Entropy	LMRT(P)	BLRT(P)	Proportion
1	20	-19253.480	38546.960	38651.058	38587.527				
2	31	-17974.103	36010.207	36171.558	36073.085	0.923	0.0000	0.0000	0.77/0.23
3	42	-17530.801	35145.602	35364.207	35230.792	0.883	0.0000	0.0000	0.24/0.55/0.20
4	53	-17181.407	34468.814	34744.673	34576.315	0.916	0.0000	0.0000	0.24/0.19/0.06/0.51
5	64	-16902.535	33933.071	34266.184	34062.884	0.925	0.0000	0.0000	0.24/0.14/0.06/0.38/0.18

k, free parameters; AIC, Akaike information criterion; BIC, Bayesian information criterion; aBIC, adjusted BIC; LMRT, Lo-Mendell-Rub test; BLRT, Bootstrap Likelihood ratio test.

Although the 4-profile and 5-profile solutions showed lower AIC, BIC, and aBIC values than the 3-profile solution, both contained a class representing only 6% of the sample. In the present study, classes of this size were considered borderline small and were interpreted cautiously, because they may reflect overextraction or subdivision of broader severity strata rather than robust and clinically meaningful groups. Moreover, inspection of the profile plots suggested that the additional classes in the 4-profile and 5-profile models mainly represented further splitting of adjacent severity levels rather than clearly distinct symptom configurations.

By contrast, the 3-profile model demonstrated acceptable entropy (0.883), adequate class proportions (24.4%, 55.5%, and 20.1%), and a parsimonious structure that was easier to interpret clinically. Thus, the 3-profile solution was retained as the optimal model, not because it had the best numerical fit on all indices, but because it offered a more balanced combination of classification quality, profile size, parsimony, and interpretability.

To further describe classification quality, the average posterior probabilities for the retained 3-profile model were examined. The correct classification probabilities for Profile 1, Profile 2, and Profile 3 were 94.2%, 95.4%, and 95.8%, respectively, indicating good separation of the retained classes (**Table 3**). However, because average posterior probabilities for the 4-profile and 5-profile solutions were not retained for formal comparison, the superiority of the 3-profile model should be interpreted primarily on the basis of parsimony, class size, and substantive interpretability rather than on posterior classification statistics alone.

**Table 3:** Attribution probabilities for each latent profile of subjects.

Class	Profile 1	Profile 2	Profile 3
Profile 1	0.942	0.058	0.000
Profile 2	0.037	0.954	0.009
Profile 3	0.000	0.042	0.958

#### 3.3.2 Characterization of the three latent depressive symptom profiles

**Figure 2:**
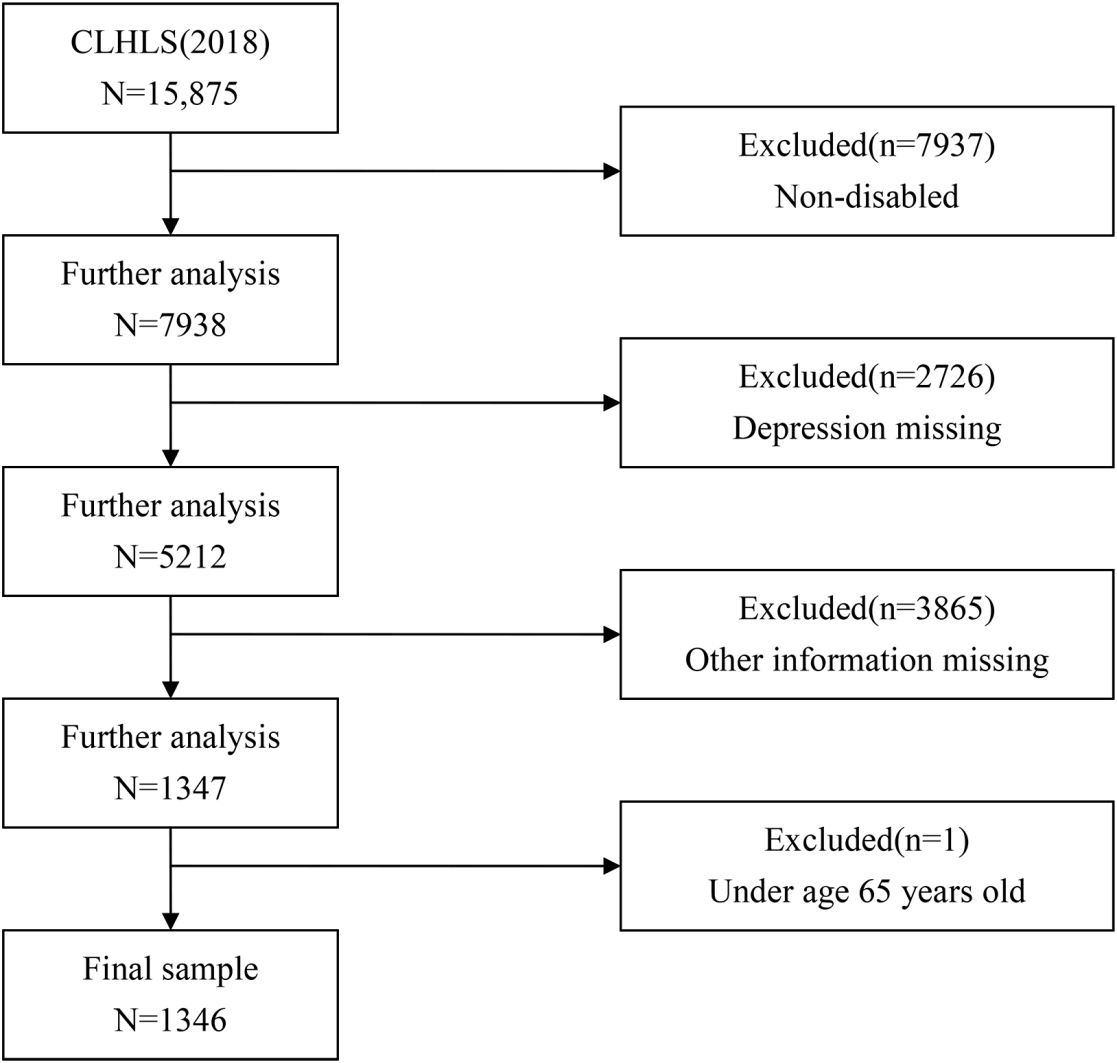
Latent profile model of depression in disabled elderly individuals.

**Figure 2** shows the item-response patterns of the three latent depressive symptom profiles among older adults with disabilities. Because the CESD-10 positive items were reverse-coded before analysis, higher scores on all items indicate greater depressive symptom burden. Accordingly, for positively worded items, higher scores should be interpreted as reflecting lower levels of positive affect or hopefulness rather than more positive emotional experiences. Profile 1 (“low-level”) showed the lowest scores across all CESD-10 items and included 24.4% of the sample. Profile 2 (“moderate-level”) showed intermediate scores across items with relatively parallel item-response patterns and comprised 55.5% of the sample. Profile 3 (“high-level”) showed the highest scores across items and comprised 20.1% of the sample. Overall, the three profiles appeared to differ primarily in the overall level of depressive symptom burden rather than in sharply distinct item-response configurations.

#### 3.3.3 Univariate analysis across latent depressive symptom profiles

To examine differences in individual correlates across depressive symptom profiles among disabled older adults, we used the chi-square test and the Kruskal-Wallis H test. Univariate analyses showed significant differences across depression profiles in gender, age, marital status, education level, co-residence status, residential area, drinking, smoking, exercise, self-rated health, activity limitations, anxiety, and MMSE, whereas serious illness was not significantly different among the profiles, as shown in **Table 4**.

**Table 4:** Univariate analysis of different latent profiles of depression in disabled elderly individuals (n [%]).

Characteristics	Low-level n=328	Moderate-level n=747	High-level n=271	χ^2^/H	P
**Gender**				χ^2^ = 16.200	**<0.001**
Male	124 (21.6)	306 (53.3)	144 (25.1)		
Female	204 (26.4)	441 (57.1)	127 (16.5)		
**Age**				χ^2^ = 18.626	**0.005**
65-75	78 (18.6)	246 (58.7)	95 (22.7)		
76-85	127 (29.7)	224 (52.5)	76 (17.8)		
86-95	75 (24.4)	163 (53.0)	70 (22.7)		
≥96	48 (25.0)	114 (59.4)	30 (15.6)		
**Marital status**				χ^2^ = 21.569	**<0.001**
No spouse	209 (28.7)	396 (54.5)	122 (16.8)		
Have a spouse	119 (19.2)	351 (56.7)	149 (24.1)		
**Education level**				χ^2^ = 29.588	**<0.001**
None	183 (30.1)	333 (54.8)	92 (15.1)		
1-6	85 (20.7)	225 (54.7)	101 (24.6)		
>6	60 (18.3)	189 (57.8)	78 (23.9)		
**Co-residence status**				χ^2^ = 19.475	**0.001**
With household member(s)	213 (21.6)	567 (57.4)	207 (21.0)		
Alone	86 (29.9)	148 (51.4)	54 (18.8)		
In an institution	29 (40.8)	32 (45.1)	10 (14.1)		
**Residential area**				χ^2^ = 55.496	**<0.001**
City	76 (16.4)	321 (69.3)	66 (14.3)		
Town	115 (28.4)	191 (47.2)	99 (24.4)		
Rural	137 (28.7)	235 (49.2)	106 (22.2)		
**Drinking**				χ^2^ = 40.871	**<0.001**
No	292 (26.0)	639 (56.9)	192 (17.1)		
Yes	36 (16.1)	108 (48.4)	79 (35.4)		
**Smoking**				χ^2^ = 12.633	**0.002**
No	288 (25.6)	628 (55.8)	209 (18.6)		
Yes	40 (18.1)	119 (53.8)	62 (28.1)		
**Exercise**				χ^2^ = 13.144	**0.001**
No	179 (25.2)	364 (51.3)	166 (23.4)		
Yes	149 (23.4)	383 (60.1)	105 (16.5)		
**Self-rated health**				χ^2^ = 18.133	**0.001**
Good	155 (25.5)	316 (52.1)	136 (22.4)		
Fair	138 (27.1)	285 (56.0)	86 (16.9)		
Bad	35 (15.2)	146 (63.5)	49 (21.3)		
**Serious illness**				χ^2^ = 3.090	0.213
None	221 (25.4)	467 (53.7)	181 (20.8)		
≥1	107 (22.4)	280 (58.7)	90 (18.9)		
**Activity limitations**				χ^2^ = 17.364	**0.002**
Strongly limited	23 (19.2)	71 (59.2)	26 (21.7)		
Limited	101 (21.4)	294 (62.2)	78 (16.5)		
Not limited	204 (27.1)	382 (50.7)	167 (22.2)		
**Anxiety**	328 (24.4)	747 (55.5)	271 (20.1)	H = 32.280	**<0.001**
**MMSE**	328 (24.4)	747 (55.5)	271 (20.1)	H = 228.918	**<0.001**

Bold values indicate statistical significance at P < 0.05.

#### 3.3.4 Multivariable analysis of factors associated with latent depressive symptom profile membership

Multinomial logistic regression analysis was performed by including variables with P < 0.05 in the univariate analysis as independent variables and the LPA classification results as the dependent variable, using the low-level and moderate-level categories as reference groups for between-group comparisons, in order to examine factors associated with depression profile membership among disabled older adults. In the retained three-profile solution, the smallest latent class contained 271 participants (20.1%), suggesting that each class had an adequate sample size for subsequent multinomial logistic regression analysis. In the regression model, the low-level and moderate-level profiles were used as reference categories for pairwise comparisons. Continuous variables (MMSE and anxiety) were entered as continuous predictors, whereas categorical variables were analyzed relative to prespecified reference groups shown in **Table 5**.

**Table 5:** Coding of study variables.

Variable	Assignment mode
Gender	Male = 1; Female = 2
Age	65–75 = 1; 76–85 = 2; 86–95 = 3; ≥96 = 4
Marital status	No spouse = 1; Have a spouse = 2
Education level	None = 1; 1–6 = 2; ≥7 = 3
Co-residence status	With household member(s) = 1; Alone = 2; In an institution = 3
Residential area	City = 1; Town = 2; Rural = 3
Drinking	Yes = 1; No = 2
Smoking	Yes = 1; No = 2
Exercise	Yes = 1; No = 2
Self-rated health	Good = 1; Fair = 2; Bad = 3
Activity limitations	Strongly limited = 1; Limited = 2; Not limited = 3
Anxiety	Measured value
MMSE	Measured value

**Table 6** presents the multinomial logistic regression results. Smoking and exercise were not significantly associated with profile membership in the adjusted models. Compared with the low-level profile, the moderate-level profile was significantly associated with MMSE, anxiety, marital status, education level, co-residence of the interviewee, residential area, drinking, smoking, exercise, self-rated health, and activity limitations. Notably, anxiety (OR = 1.197, P<0.001), activity limitations (OR = 1.373, P = 0.047), living with household members (OR = 2.906, P<0.001), and urban residency (OR = 2.354, P<0.001) were associated with membership in the moderate-level profile. Compared with the low-level profile, the high-level profile was significantly associated with MMSE, anxiety, gender, age, marital status, education level, Co-residence status, drinking, and self-rated health. Specifically, anxiety (OR = 1.235, P<0.001), male (OR = 1.924, P = 0.002), age group 65–75 years (OR = 2.025, P = 0.036), living with household members (OR = 5.153, P = 0.001), and drinking (OR = 3.305, P<0.001) were associated with membership in the high-level profile. In addition, compared with the moderate-level profile, membership in the high-level profile was significantly associated with MMSE, gender, marital status, residential area, drinking, and activity limitations. Among these factors, male gender (OR = 1.653, P = 0.004) and drinking (OR = 2.340, P < 0.001) were associated with membership in the high-level profile.

**Table 6:** Multivariate logistic regression analysis of different potential factors in disabled elderly individuals.

Reference group	Low-level						Moderate-level		
Comparison group	Moderate-level			High-level			High-level		
	OR	95%CI	P	OR	95%CI	P	OR	95%CI	P
Gender									
Male	1.164	0.871-1.554	0.305	1.924	1.281-2.888	**0.002**	1.653	1.174-2.328	**0.004**
Age									
65-75	1.331	0.845-2.098	0.218	2.025	1.048-3.913	**0.036**	1.521	0.873-2.650	0.139
76-85	0.748	0.483-1.157	0.192	0.901	0.465-1.746	0.757	1.204	0.677-2.144	0.527
86-95	0.879	0.551-1.403	0.589	1.479	0.746-2.934	0.262	1.684	0.933-3.036	0.084
Marital status									
No spouse	0.615	0.461-0.822	**0.001**	0.397	0.264-0.596	**<0.001**	0.644	0.457-0.908	**0.012**
Education level									
None	0.540	0.374-0.782	**0.001**	0.390	0.233-0.652	**<0.001**	0.722	0.472-1.104	0.132
1-6	0.782	0.518-1.180	0.241	0.946	0.551-1.624	0.840	1.210	0.786-1.861	0.386
Co-residence status									
With household member(s)	2.906	1.637-5.158	**<0.001**	5.153	1.882-14.107	**0.001**	1.774	0.687-4.577	0.236
Alone	1.652	0.888-3.075	0.113	2.881	0.990-8.383	0.052	1.744	0.640-4.750	0.277
Residential area									
City	2.354	1.637-3.385	**<0.001**	1.261	0.748-2.128	0.384	0.536	0.347-0.828	**0.005**
Town	0.844	0.601-1.184	0.326	1.030	0.639-1.659	0.904	1.221	0.808-1.845	0.344
Drinking									
Yes	1.412	0.921-2.165	0.113	3.305	1.950-5.601	**<0.001**	2.340	1.555-3.521	**<0.001**
Smoking									
Yes	1.331	0.877-2.022	0.180	1.545	0.902-2.647	0.113	1.160	0.757-1.779	0.495
**Exercise**									
Yes	1.012	0.754-1.360	0.936	0.737	0.485-1.122	0.155	0.729	0.510-1.040	0.081
Self-rated health									
Good	0.550	0.343-0.882	**0.013**	0.599	0.312-1.150	0.123	1.089	0.646-1.835	0.750
Fair	0.599	0.377-0.952	**0.030**	0.517	0.272-0.984	**0.044**	0.863	0.516-1.443	0.574
Activity limitations									
Strongly limited	1.303	0.753-2.256	0.345	0.998	0.471-2.118	0.996	0.766	0.417-1.406	0.390
Limited	1.373	1.005-1.876	**0.047**	0.858	0.543-1.356	0.512	0.625	0.425-0.920	**0.017**
**Anxiety**	1.197	1.113-1.288	**<0.001**	1.235	1.131-1.350	**<0.001**	1.032	0.973-1.094	0.219
**MMSE**	0.917	0.884-0.951	**<0.001**	0.746	0.714-0.778	**<0.001**	0.813	0.789-0.837	**<0.001**

Bold values indicate statistical significance at P < 0.05.

## 4 Discussion

This study extends the literature on late-life depression by identifying three latent depressive symptom profiles among Chinese older adults with disabilities. Rather than assuming a uniform pattern of depression in this population, the findings suggest meaningful heterogeneity in symptom presentation. This person-centered approach may help refine mental health assessment in disabled older adults by showing that depressive symptoms cluster at different severity levels. However, the findings should be interpreted cautiously. Because this study was based on cross-sectional and largely self-reported data, the observed relationships should be understood as associative rather than causal. In addition, some potentially relevant variables, such as comorbidities, medication use, and financial hardship, were not available in the dataset and may have contributed to the observed between-profile difference.

### 4.1 Characteristics of the latent depression profiles in disabled older adults

This study identified three distinct profiles of depressive symptoms: “low-level, “ “moderate-level, “ and “high-level.” The delineation of these subtypes indicated heterogeneity in depressive symptom burden within the disabled older adult population. A substantial proportion of participants (75.6%) were classified into the moderate-level or high-level profiles. This finding suggests that depressive symptoms were common in this population and is generally consistent with previous research showing that older adults with physical disability and social disadvantage may report higher levels of depressive symptoms (35). However, because this study was based on cross-sectional screening data, these profiles should be understood as reflecting differences in symptom burden rather than clinically confirmed severity categories.

Low-Level Depression: Disabled older adults in the low-level profile had relatively low scores across CESD-10 items overall, although several items were comparatively higher than others within the same profile. Item 7, “Are you as happy as when you were younger?”, showed the highest relative score. Because this is a positively worded item that was reverse-coded, a higher score indicates reduced positive affect in comparison with earlier life, rather than directly measuring constructs such as nostalgia or psychological conflict (36). Item 10, “How about the quality of your sleep?” was also relatively high, indicating that sleep quality is universally affected in this group. Research suggests a sophisticated interplay between sleep disturbances and depressive symptoms, where chronic sleep disruptions elevate the likelihood of developing depression (37). Furthermore, elements like physical discomfort, pharmacological side effects, and psychological comorbidities—including anxiety and depressive disorders—also play a role in the genesis of sleep disturbances. Item 5, “Do you feel hopeful about the future?” also showed a relatively higher score. Because this item is positively worded and reverse-coded in the CESD-10, a higher score should be interpreted as reflecting lower hopefulness about the future rather than a directly measured construct such as pessimism. Despite the overall low level of depressive symptoms, the relatively higher scores on specific items suggest that these aspects may merit closer descriptive attention in future assessment and research.

Moderate-Level Depression: In this study, 55.5% of disabled older adults were classified into the moderate-level profile, representing the largest group. Item 7, “Are you as happy as when you were younger?”, remained relatively higher than most other items. Given the reverse coding of this positively worded item, this result is more appropriately interpreted as indicating reduced positive affect related to one’s current life situation, rather than a directly measured discrepancy between past and present life quality. Item 5, “Do you feel hopeful about the future?”, also showed a relatively higher score. Given the reverse coding of positively worded CESD-10 items, this finding indicates lower self-reported hopefulness about the future. However, broader interpretations such as pessimism, uncertainty, or perceived loss of independence were not directly measured in the present study and therefore should be interpreted cautiously. The relatively greater prominence of these items suggests that reduced positive affect and lower hopefulness may be more salient features within this profile. Individuals in this group may warrant closer monitoring in future studies, although whether early identification or additional support would alter subsequent depressive symptom trajectories remains to be established.

High-Level Depression: In this study, 20.1% of disabled older adults were classified into the high-level profile, which showed the greatest overall depressive symptom burden. Scores on Item 4, “Do you feel the older you get, the more useless you are, and have trouble doing anything?”, Item 7, “Are you as happy as when you were younger?”, and Item 10, “How about the quality of your sleep?” were all above 2.4 points. These relatively higher scores suggest that negative self-appraisal, reduced positive affect, and sleep-related symptoms were more prominent in this profile. The elevated score on Item 4 should be interpreted as indicating stronger endorsement of feeling less useful and having difficulty doing things, rather than as evidence of a specific psychological mechanism such as reduced self-efficacy. Similarly, the higher score on the item comparing current happiness with earlier life may reflect reduced positive affect in relation to one’s current life situation, although the underlying psychological meaning of this response cannot be fully determined from the present dataset (38). Overall, this profile was characterized by a higher symptom burden across CESD-10 items, with relatively greater prominence of self-worth-related, affective, and sleep-related symptoms. These findings may help identify domains that deserve further attention in future assessment and research, but they do not by themselves establish which type of support would be most effective.

### 4.2 Associated factors of potential profiles of depression in disabled elderly individuals

#### 4.2.1 Demographic characteristics factors

Exploring demographic correlates of depressive symptom profile membership among older adults with disabilities may help inform future screening and supportive care. In this study, gender, age, marital status, education level, co-residence, residential area, alcohol consumption, and self-rated health were associated with profile membership. However, because the study was cross-sectional, these findings should be interpreted as associations rather than as evidence of causal pathways or underlying mechanisms.

Gender: In the adjusted analysis, male sex was more frequently associated with membership in the high-level depressive symptom profile (39). This finding is consistent with some previous studies reporting gender differences in depressive symptoms among older adults with functional limitations. However, the present study did not assess biological factors, gender-role expectations, or coping styles. Therefore, the reasons underlying this association cannot be determined from the current data. The observed gender difference should be interpreted cautiously as an association that may warrant further investigation in future longitudinal studies. These findings suggest that male older adults with disabilities may warrant closer attention in future mental health screening and supportive care.

Age: Age was also associated with depressive symptom profile membership among disabled older adults. This finding is broadly consistent with previous research showing that depressive symptoms may vary across age groups in later life (40). However, the current study did not directly examine disease burden, functional decline trajectories, bereavement, or retirement-related social changes. Therefore, the age-related findings should be interpreted cautiously as descriptive associations rather than as evidence that age itself leads to more severe depressive symptoms.

Marital Status: This research uncovered a substantial association between marital status and depressive symptom profile membership. In this sample, older adults without a spouse were less likely to belong to the more severe depressive symptom profiles than those with a spouse, which differs from some prior findings. This discrepancy should be interpreted with caution. The present study did not assess relationship quality, caregiving burden, duration of widowhood, social support, or psychological resilience, and therefore cannot determine why this association was observed. This finding may indicate the need for further attention to relationship context and perceived caregiving burden when assessing mental health among disabled older adults (41).

Education Level: Education level was associated with depressive symptom profile membership in the adjusted model. Although participants with formal education appeared more likely to belong to more severe profiles in some comparisons, this finding should be interpreted cautiously. The current dataset did not include measures of expectations, perceived role loss, coping style, or other psychosocial processes that might account for this association. Therefore, the observed relationship should be understood as a statistical association rather than as evidence that higher education increases depressive symptom risk (42).

Co-Residence: Co-residence was also associated with depressive symptom profile membership. Older adults living with family members showed a higher likelihood of belonging to more severe profiles than those living alone in some comparisons (43). However, this finding should not be interpreted to mean that co-residence itself worsens depressive symptoms. Because the present study did not measure family relationship quality, caregiving strain, household conflict, or perceived support, the mechanisms underlying this association remain unclear. This finding may suggest that the quality of family support, rather than co-residence itself, is more closely related to depressive symptom patterns in this population.

Residential Area: Residential area was associated with depressive symptom profile membership, with urban residence showing a stronger association with more severe profiles in some comparisons. This finding should be interpreted cautiously. Although differences in social environment, access to services, family structure, or lifestyle may contribute to urban-rural variation, these factors were not directly measured in the present study. Therefore, the observed urban-rural difference cannot be attributed to specific mechanisms such as migration, lifestyle change, or social isolation on the basis of the current analysis alone (44–46).

Alcohol Consumption: Alcohol consumption was associated with depressive symptom profile membership in the adjusted model (47, 48). However, because this study did not assess drinking frequency, quantity, duration, or alcohol-related health consequences, the meaning of this association should be interpreted cautiously. The present findings do not allow conclusions about whether alcohol use contributes to depressive symptoms, whether depressive symptoms influence drinking behavior, or whether both are shaped by other unmeasured factors. The observed association suggests that alcohol use may be an important factor to consider in future mental health assessment among disabled older adults.

Self-Rated Health: Self-rated health status was significantly associated with depressive symptom profile membership (49). Although self-rated health is a subjective evaluation, it may reflect how older adults perceive their overall physical and mental condition. In the present study, poorer self-rated health was associated with membership in more severe depressive symptom profiles. However, because self-rated health is a broad subjective indicator and the study was cross-sectional, this association should not be interpreted as evidence of directionality. Previous studies have shown that self-rated health is closely related to overall health status in later life (50), and better self-rated health has been associated with greater well-being and lower levels of depressive symptoms (51). Conversely, poorer self-rated health may coexist with functional burden, stress, depressive symptoms, and other unmeasured clinical conditions (52). Addressing the mental health of disabled older adults may therefore require a comprehensive consideration of physical, psychological, and social factors.

#### 4.2.2 Anxiety

Anxiety was closely associated with depressive symptom profile membership in this study. This finding is consistent with previous literature showing substantial overlap between anxiety and depressive symptoms among older adults, including those with disability (53). However, because the present study was cross-sectional, it cannot determine whether anxiety precedes depressive symptoms, follows them, or co-occurs due to shared underlying vulnerabilities. In addition, although disabled older adults may face mobility limitations, social restriction, chronic pain, and reduced independence, these pathways were not directly tested in the current analysis (54, 55). Therefore, anxiety should be interpreted as an important correlate, rather than a confirmed antecedent or mechanism, of depressive symptom profile membership. Given the close association observed in this study, anxiety symptoms may be an important factor to consider in future screening and supportive care for disabled older adults.

#### 4.2.3 MMSE

Better cognitive function was associated with lower levels of depressive symptom profile membership in this study (56). This finding is in line with previous research suggesting a close relationship between cognitive status and mental health in older adults (57, 58). However, the present study cannot determine the direction of this association. Cognitive impairment may coexist with depressive symptoms, and both may also be related to broader health decline, disability severity, or other unmeasured factors. Therefore, cognitive status should be interpreted as an important correlate of depressive symptom profiles rather than as evidence of a direct protective mechanism. Nevertheless, the present findings suggest that cognitive status may be an important factor to consider when evaluating mental health in disabled older adults.

## 5 Strengths, limitations, and future directions

Our study is the first to apply latent profile analysis to depressive symptoms among older adults with disabilities in China and provides a person-centered description of symptom heterogeneity in this population. Nevertheless, several limitations should be noted. First, this study used cross-sectional data from a single time point, so the observed associations cannot be interpreted as causal, and the temporal ordering between variables could not be established. Second, the final analytical sample was derived using a complete-case approach, and a considerable number of participants were excluded because of missing information on depression, disability status, or other study variables. We did not conduct a formal analysis of missing-data patterns or compare the characteristics of included and excluded participants. Therefore, selection bias cannot be ruled out, particularly because missingness in very old adults with disability may be related to health status, cognitive impairment, or survey response capacity rather than occurring completely at random. Third, most variables were based on self-report, which may have introduced recall bias and reporting bias. Although Harman’s single-factor test suggested that severe common method bias was not evident, this method is limited and is generally considered a relatively weak diagnostic tool. It cannot fully rule out bias related to self-report measures, shared measurement context, or the cross-sectional nature of the data. Fourth, because this study relied on a public database, some potentially important variables were unavailable, such as comorbid health conditions, medication use, and financial difficulties. These unmeasured factors may have influenced depression profile membership and may partly explain the observed between-group differences. Future studies should use more rigorous missing-data handling strategies, such as detailed missingness diagnostics and, where appropriate, multiple imputation, and should include more comprehensive clinical, treatment-related, and socioeconomic variables to better clarify the mechanisms underlying depressive symptoms in older adults with disabilities. In addition, because disability was operationally defined using dependence in any BADL or IADL item, the study sample may still have included individuals with different levels of functional severity, and the findings should be interpreted with this heterogeneity in mind.

## 6 Conclusions

Three latent depressive symptom profiles were identified among Chinese older adults with disabilities, and several demographic, health-related, and psychosocial factors were associated with profile membership. The retained profiles appeared to differ primarily in symptom severity rather than in clearly distinct subtypes. Therefore, these findings should be interpreted as descriptive evidence of heterogeneity in depressive symptom burden, and they may provide a basis for future longitudinal, prognostic, and intervention research rather than direct evidence for profile-specific intervention design.

## Data Availability

The raw data supporting the conclusions of this article will be made available by the authors, without undue reservation.
